# Diminished Reovirus Capsid Stability Alters Disease Pathogenesis and Littermate Transmission

**DOI:** 10.1371/journal.ppat.1004693

**Published:** 2015-03-04

**Authors:** Joshua D. Doyle, Jennifer E. Stencel-Baerenwald, Courtney A. Copeland, Jillian P. Rhoads, Judy J. Brown, Kelli L. Boyd, James B. Atkinson, Terence S. Dermody

**Affiliations:** 1 Department of Pathology, Microbiology, and Immunology, Vanderbilt University School of Medicine, Nashville, Tennessee, United States of America; 2 Elizabeth B. Lamb Center for Pediatric Research, Vanderbilt University School of Medicine, Nashville, Tennessee, United States of America; 3 Department of Pediatrics, Vanderbilt University School of Medicine, Nashville, Tennessee, United States of America; MFP CNRS UMR 5234 at University Bordeaux 2, FRANCE

## Abstract

Reovirus is a nonenveloped mammalian virus that provides a useful model system for studies of viral infections in the young. Following internalization into host cells, the outermost capsid of reovirus virions is removed by endosomal cathepsin proteases. Determinants of capsid disassembly kinetics reside in the viral σ3 protein. However, the contribution of capsid stability to reovirus-induced disease is unknown. In this study, we found that mice inoculated intramuscularly with a serotype 3 reovirus containing σ3-Y354H, a mutation that reduces viral capsid stability, succumbed at a higher rate than those infected with wild-type virus. At early times after inoculation, σ3-Y354H virus reached higher titers than wild-type virus at several sites within the host. Animals inoculated perorally with a serotype 1 reassortant reovirus containing σ3-Y354H developed exaggerated myocarditis accompanied by elaboration of pro-inflammatory cytokines. Surprisingly, unchallenged littermates of mice infected with σ3-Y354H virus displayed higher titers in the intestine, heart, and brain than littermates of mice inoculated with wild-type virus. Together, these findings suggest that diminished capsid stability enhances reovirus replication, dissemination, lethality, and host-to-host spread, establishing a new virulence determinant for nonenveloped viruses.

## Author Summary

Following attachment and internalization, viruses disassemble to complete the entry process, establish infection, and cause disease. Viral capsid stability balances on a fulcrum, as viruses must be sufficiently stable in the environment to reach the host yet also uncoat efficiently once the target cell barrier has been breached. Reoviruses are useful models to understand the relationship between viral entry and pathogenesis. Residues within reovirus outer-capsid protein σ3 influence capsid stability, but the function of capsid stability in disease pathogenesis was not known. We found that serotype 1 and serotype 3 reovirus variants with diminished capsid stability attributable to a single amino change in σ3 displayed enhanced lethality in newborn mice following peroral and intramuscular inoculation, respectively. In the serotype 1 background, this variant caused increased damage to cardiac tissue and increased elaboration of inflammatory mediators in comparison to wild-type virus. Remarkably, diminished capsid stability also enhanced the spread of virus between inoculated and uninoculated littermates. Taken together, these findings define a new virulence determinant for reovirus and shed light on general principles of viral pathogenesis for nonenveloped viruses.

## Introduction

Penetration of a target cell membrane is an essential requirement for infection by all viruses. Enveloped viruses use structural rearrangements of specialized proteins to drive fusion of the viral envelope and host membranes. Fusion events can occur at the cell surface, as with most retroviruses [1,2], or at an endosomal membrane following internalization, as with alphaviruses [3–5], filoviruses [6,7], orthomyxoviruses [8,9], and paramyxoviruses [10–12]. In contrast, nonenveloped viruses often encode proteins that disrupt host membranes in response to cues imparted by target cells, such as receptor binding, endosomal acidification, or proteolytic cleavage. In some instances, as with picornaviruses, the same capsid components are involved in both receptor engagement and membrane penetration [13–16]. For other viruses, including adenoviruses and reoviruses, receptor binding and membrane bypass occur at different cellular sites and are mediated by distinct viral proteins [17–21]. Structural rearrangement or partial disassembly of the virion is often required to promote interactions with a target cell membrane. Capsid structures must thus carefully balance a demand for stability sufficient for environmental persistence with instability required for the conformational alterations necessary for infection. However, despite its importance in cell entry and replication, the contribution of capsid stability to viral pathogenesis is poorly understood.

Mammalian orthoreoviruses (or simply reoviruses) are nonenveloped viruses with an extremely broad natural host range [22,23]. Reovirus virions are composed of two concentric protein shells, an outer capsid, formed primarily by viral proteins σ3 and μ1, and a transcriptionally active inner core. The inner core contains the viral genome, which consists of ten segments of double-stranded RNA. Virtually all mammals are hosts for reovirus infection with transmission occurring readily between mammalian species [23,24]. Reoviruses are not associated with significant human disease, but infections of newborn mice lead to systemic viral replication, morbidity, and mortality. Reovirus strains exhibit serotype-specific dissemination and tissue tropism. Following peroral inoculation, type 1 reovirus strains are taken up by Peyer’s patches in the intestine [25,26] and disseminate via hematogenous routes [27,28]. Type 3 strains are neurotropic, disseminate via neural routes, and produce CNS injury [29–31]. Mice infected with some type 3 reoviruses also develop hepatobiliary pathology [32], and epidemiological evidence suggests a relationship between reovirus and human neonatal biliary atresia [33–36]. Reovirus produces enhanced cytotoxicity in cancer cell lines [37–39] and causes tumor regression in mouse xenografts [37]. There are ongoing studies to evaluate the efficacy of reovirus (under the trade name Reolysin) as a clinical antineoplastic agent.

The initial step in reovirus cell entry is the binding of attachment protein σ1 to cell-surface receptors, including glycans [40,41] and junctional adhesion molecule-A (JAM-A) [42–45]. Following receptor engagement, reovirus virions are internalized via clathrin-mediated endocytosis in a process dependent on β1 integrin [46] and transported to late endosomes marked by Rab GTPases 7 and 9 [46,47]. The acidic environment of late endosomes activates resident endosomal proteases, including cathepsin family members [48]. In particular, cathepsins B and L mediate stepwise disassembly of the reovirus outer capsid beginning with proteolysis and loss of σ3 protein and subsequent cleavage of μ1 protein to generate the species μ1N, μ1-δ, and μ1-φ [49–54]. These cleavage fragments disrupt endosomal membranes and afford the transcriptionally active viral core access to the cytosol. Proteolytic cleavage of σ3 is the initial and rate-limiting step of reovirus capsid disassembly.

Polymorphisms in σ3 alter its susceptibility to cellular proteases. In particular, a tyrosine-to-histidine mutation at residue 354 near the C-terminus of σ3 substantially enhances its sensitivity to cleavage by a variety of proteases. The σ3-Y354H mutation was first identified in viruses isolated from persistently infected cells, which display altered cathepsin expression [55,56]. This mutation also is selected in response to other protease-limiting conditions, including treatment with the pan-cysteine protease inhibitor, E64 [57], or ammonium chloride [58], each of which blocks acid-dependent proteolytic disassembly. Viruses containing σ3-Y354H undergo more rapid disassembly when treated with proteases *in vitro* and escape endosomes of infected cells more rapidly than viruses with wild-type σ3 [59,60]. The presence of σ3-Y354H also diminishes the thermodynamic stability of reovirus virions, causing more rapid loss of infectious titer at elevated temperature [60]. Interestingly, σ3-Y354H is not found in circulating strains of reovirus except in the setting of a suppressive glycine-to-glutamate polymorphism at position 198 in σ3 [60,61]. The limited natural occurrence of σ3-Y354H suggests that diminished outer-capsid stability imposes a fitness penalty at some stage of the viral replication cycle. This conclusion is consistent with the idea that optimal viral capsid stability is a biochemical property determined by offsetting selection pressures.

In this study, we tested the hypothesis that outer-capsid stability influences the pathogenesis of reovirus-mediated disease using reovirus strain type 3 Dearing (T3D) and a T3D mutant containing the σ3-Y354H mutation. We found that mice inoculated in the hindlimb with T3D-σ3Y354H succumbed more quickly and at higher frequency than those inoculated with wild-type T3D. Furthermore, at early time points T3D-σ3Y354H reached higher viral titers at sites of secondary replication than did T3D. We also generated reassortant reovirus strains incorporating T3D outer-capsid proteins μ1 and σ3, with and without σ3-Y354H, into the genetic background of strain type 1 Lang (T1L) for use in peroral inoculation studies. The T1L reassortant containing σ3-Y354H caused pronounced cardiac damage in infected animals and produced greater lethality than the corresponding wild-type strain. Finally, using these reassortant strains, we observed that σ3-Y354H enhances host-to-host spread between infected animals and naïve littermates. Together, these results suggest that the rate of capsid disassembly modulates reovirus pathogenesis in an unanticipated way: diminished stability enhances virulence and host-to-host transmission. These findings indicate that biochemical properties of nonenveloped viral capsids can dramatically influence disease.

## Results

### Diminished reovirus outer-capsid stability correlates with enhanced lethality

**Fig 1:**
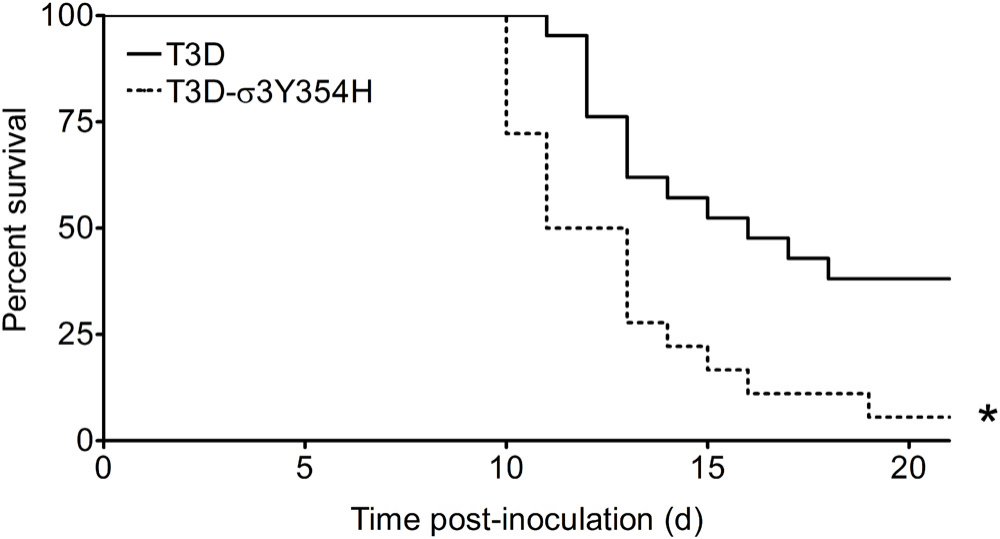
The σ3-Y354H mutation enhances reovirus virulence following intramuscular inoculation. Newborn C57BL/6J mice were inoculated in the left hindlimb with 10^5^ PFU of T3D or T3D-σ3Y354H. Mice (n = 18 and 21 for T3D and T3D-σ3Y354H, respectively) were monitored for survival for 21 days. *, *P* < 0.001 as determined by log-rank test in comparison to T3D.

To investigate whether differences in outer-capsid stability influence reovirus pathogenesis, we inoculated newborn mice in the left hindlimb with 10^5^ PFU of either wild-type T3D or protease-hypersensitive mutant T3D-σ3Y354H and monitored infected animals for survival (Fig 1). Surprisingly, a significantly higher percentage of mice inoculated with T3D-σ3Y354H succumbed to infection than those inoculated with T3D. The median survival interval for mice infected with T3D was approximately 4 days longer than that observed for animals infected with T3D-σ3Y354H. Type 3 reovirus strains are neurotropic, inducing lethal encephalitis in infected newborn mice [29,62,63]. Accordingly, animals infected with either T3D or T3D-σ3Y354H displayed neurological signs, including bilateral flaccid paralysis, dyskinesias, myoclonic jerks, and occasional seizures.

### Mice infected with T3D-σ3Y354H have increased viral loads at early times after infection

**Fig 2:**
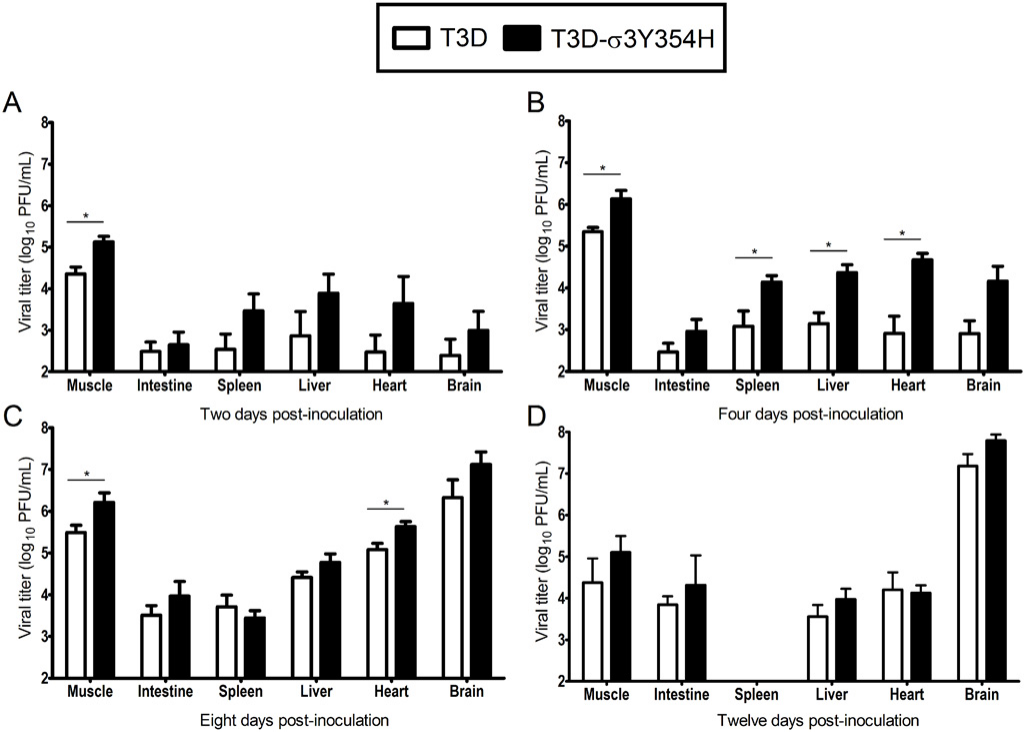
Viral loads are higher in mice infected with T3D-σ3Y354H. Newborn C57BL/6J mice were inoculated in the left hindlimb with 10^5^ PFU of either T3D or T3D-σ3Y354H. At days 2 (A), 4 (B), 8 (C), and 12 (D) post-inoculation, animals were euthanized, left hindlimb muscle, intestine, spleen, liver, heart, and brain were excised, and viral titers in organ homogenates were determined by plaque assay. Results are expressed as mean viral titers for 6 to 9 animals for each time point. Error bars indicate standard errors of the means. *, *P* < 0.05 as determined by Mann-Whitney test in comparison to T3D.

Based on the observed differences in mortality, we hypothesized that T3D-σ3Y354H replicates more rapidly *in vivo* than does T3D, resulting in more rapid dissemination and enhanced systemic disease. To test this hypothesis, newborn mice were inoculated intramuscularly with 10^5^ PFU of either T3D or T3D-σ3Y354H. Mice were euthanized at days 2, 4, 8, and 12 post-inoculation, and titers of reovirus in the left hindlimb muscle, intestine, spleen, liver, heart, and brain were determined by plaque assay (Fig 2). Animals inoculated with T3D-σ3Y354H had significantly higher viral titers in the hindlimb muscle at days 2, 4, and 8 post-inoculation than those infected with T3D. Additionally, titers of T3D-σ3Y354H were higher at days 2 and 4 post-inoculation at several sites of secondary replication, including the spleen, liver, and heart. However, by day 8 post-inoculation, differences in titers of T3D and T3D-σ3Y354H had largely dissipated and, by day 12 post-inoculation, there were no significant differences in the viral loads produced by the two viruses. Viral titers in the spleen on day 12 post-inoculation were below the limit of detection. These data suggest that σ3-Y354H facilitates more rapid initial replication in infected hosts, resulting in enhanced systemic spread.

### Construction of T1L × T3D reassortant viruses

**Fig 3:**
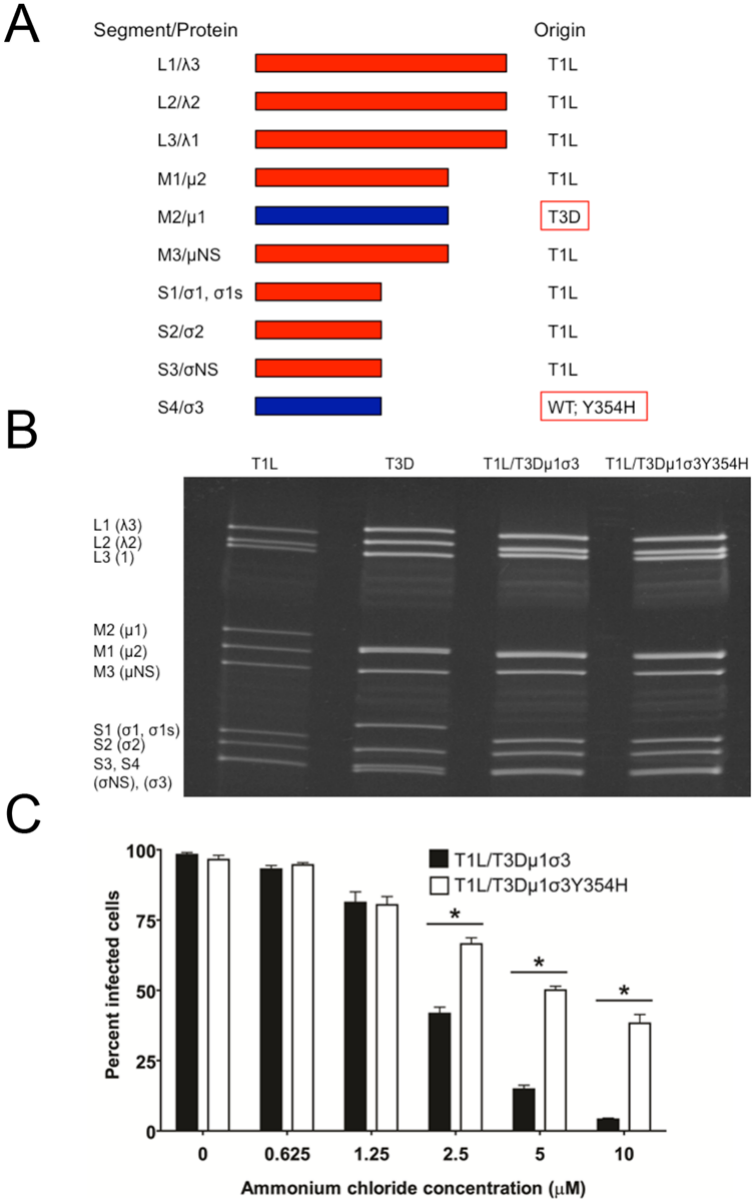
Construction of T1L × T3D reassortant reovirus strains. (A) Schematic of the genomes of the reassortant reovirus strains used in this study. Gene segments derived from T1L and T3D are shown in red and blue, respectively. Two reassortant strains were recovered by reverse genetics, incorporating either the wild-type T3D S4 gene segment encoding σ3 or T3D σ3-Y354H. (B) Electrophoretic analysis of the dsRNA genomes of recombinant reassortant viruses. Purified virions of T1L, T3D, T1L/T3Dμ1σ3, and T1L/T3Dμ1σ3Y354H were electrophoresed in an SDS-polyacrylamide gel, which was stained with ethidium bromide to visualize viral gene segments. Gene segments are labeled on the left. (C) Ammonium chloride sensitivity of reassortant viruses. Murine L929 cells were pretreated with the concentrations of ammonium chloride shown, adsorbed with T1L/T3Dμ1σ3 or T1L/T3Dμ1σ3Y354H at an MOI of 25 PFU/cell, and incubated for 18 h. Cells were fixed with methanol, and reovirus-infected cells were quantified for three independent experiments. Error bars indicate standard errors of the means. *, *P* < 0.05 as determined by Mann-Whitney test in comparison to T3D.

The Y354H mutation in σ3 is selected using a variety of conditions in cell culture [56–58]. However, this mutation is absent from circulating reovirus strains except in the presence of suppressive second-site mutations [60,61]. Enhanced susceptibility to proteolytic disassembly might diminish viability in the intestinal lumen following peroral inoculation or reduce host-to-host transmission of reovirus, limiting the prevalence of σ3-Y354H. Reovirus infection in nature is thought to occur primarily by a fecal-oral route [64,65]. However, strain T3D does not efficiently transit the digestive tract, as the T3D σ1 attachment protein is cleaved by intestinal proteases [66,67]. Therefore, to test the effect of the σ3-Y354H mutation on systemic dissemination from the intestine and transmission between littermates, we constructed reassortant viruses containing eight gene segments from strain T1L and the M2 and S4 gene segments, which encode outer-capsid proteins μ1 and σ3, respectively, from T3D (Fig 3A). The T3D M2 and S4 alleles were included together in the reassortant T1L/T3D viruses to preserve optimum interactions between σ3 and μ1 and their synergistic functions in reovirus disassembly and endosomal escape [68]. Two versions of this reassortant virus were constructed, T1L/T3Dμ1σ3Y354H and T1L/T3Dμ1σ3, respectively with and without the σ3-Y354H mutation. Genotypes of the reassortant viruses were verified following electrophoresis of viral genomic RNA (Fig 3B). In comparison to T1L/T3Dμ1σ3, T1L/T3Dμ1σ3Y354H was less sensitive to inhibition by ammonium chloride (Fig 3C), suggesting that σ3-Y354H accelerates viral disassembly in the T1L genetic background. Moreover, viruses containing σ3-Y354H were less resistant to heat than their wild-type counterparts (S1 Fig), although differences in heat-resistance were greater for wild-type and mutant strains in the genetic background of T3D than T1L. Particle-to-PFU ratios of two independent stocks of T1L/T3Dμ1σ3 and T1L/T3Dμ1σ3Y354H averaged 148:1 and 163:1, respectively. This value is consistent with those previously reported for reoviruses [69] and suggests that any differences displayed by these strains are not attributable to alterations in specific infectivity.

### The Y354H mutation in σ3 enhances lethality of reovirus following peroral challenge

**Fig 4:**
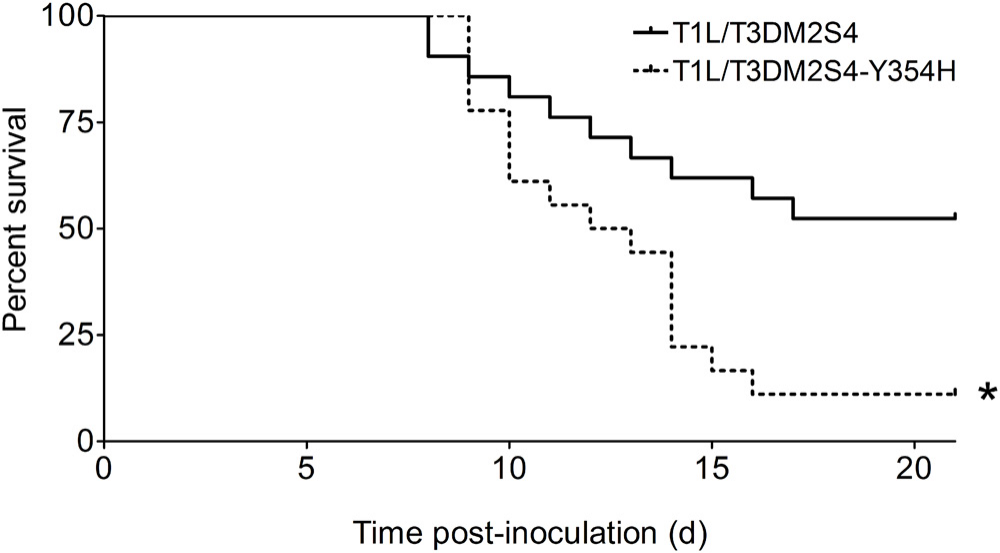
A reassortant reovirus strain containing σ3-Y354H displays enhanced virulence following peroral inoculation. Newborn C57BL/6J mice were inoculated perorally with 10^4^ PFU of either T1L/T3Dμ1σ3 or T1L/T3Dμ1σ3Y354H. Mice (n = 21 and 18 for T1L/T3Dμ1σ3 and T1L/T3Dμ1σ3Y354H, respectively) were monitored for survival for 21 days. *, *P* < 0.001 as determined by log-rank test in comparison to T1L/T3Dμ1σ3.

To determine whether capsid stability affects reovirus virulence following a natural route of infection, newborn mice were inoculated perorally with 10^4^ PFU of either reassortant strain T1L/T3Dμ1σ3 or T1L/T3Dμ1σ3Y354H and monitored for survival (Fig 4). Similar to results gathered using T3D and T3Dσ3-Y354H, a significantly higher percentage of mice inoculated with T1L/T3Dμ1σ3Y354H succumbed to infection in comparison to those inoculated with T1L/T3Dμ1σ3. The reassortant strains express a serotype 1 σ1 attachment protein, which promotes efficient systemic spread but does not mediate neural transmission or infection of CNS neurons [27,28,31]. Accordingly, infected animals displayed lethargy beginning 8 days post-inoculation that progressed throughout the observation interval, but neurological findings were absent in mice infected with either reassortant strain.

**Fig 5:**
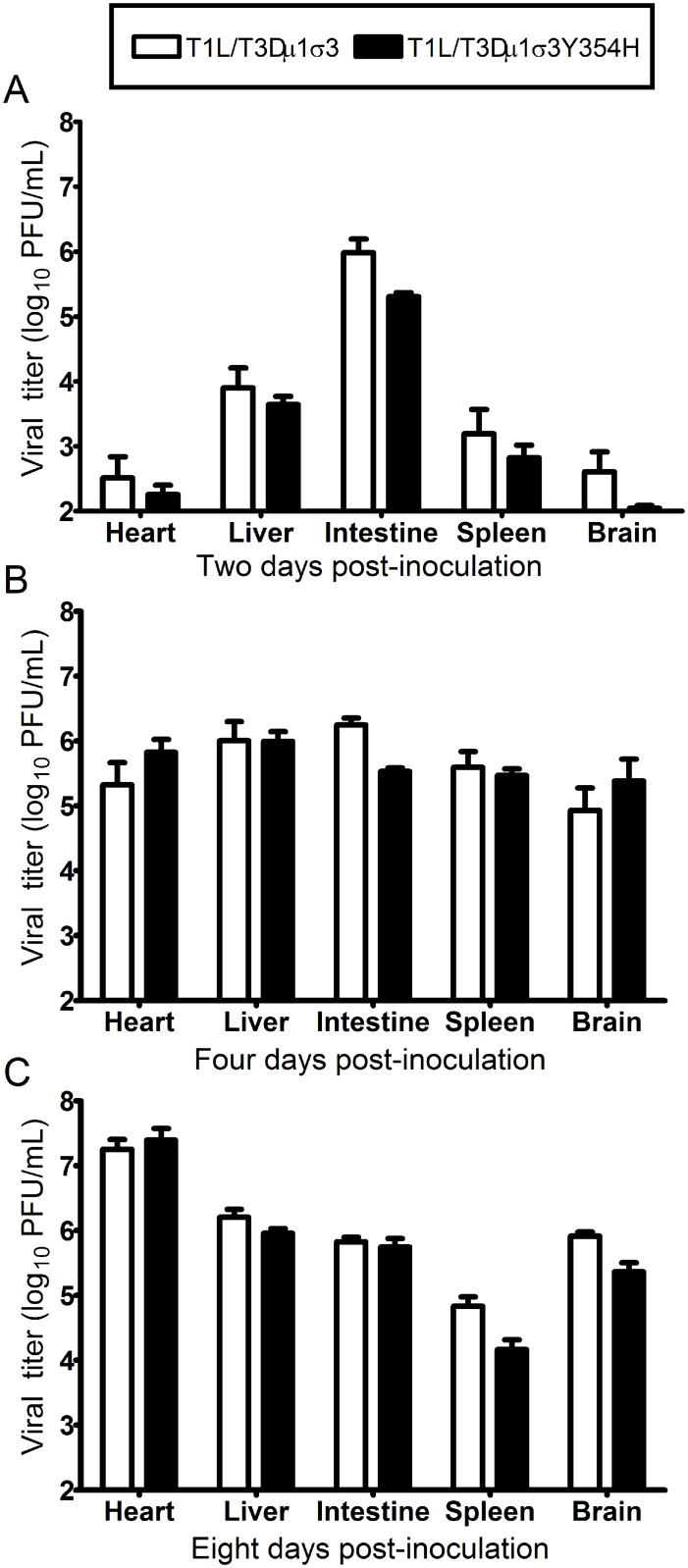
Viral loads are comparable in mice infected with reassortant viruses. Newborn C57BL/6J mice were inoculated perorally with 10^4^ PFU of either T1L/T3Dμ1σ3 or T1L/T3Dμ1σ3Y354H. At days 2 (A), 4 (B), 8 (C) post-inoculation, animals were euthanized, intestine, spleen, liver, heart, and brain were excised, and viral titers in organ homogenates were determined by plaque assay. Results are expressed as mean viral titers for 6 to 9 animals for each time point. Error bars indicate standard errors of the mean.

We hypothesized that the enhanced lethality of T1L/T3Dμ1σ3Y354H compared with T1L/T3Dμ1σ3 might be due to differences in initial replication or dissemination between the two viruses. We inoculated newborn mice perorally with 10^4^ PFU of either T1L/T3Dμ1σ3 or T1L/T3Dμ1σ3Y354H, harvested intestine, spleen, liver, heart, and brain at days 2, 4, and 8 post-inoculation, and determined viral titers in those organs by plaque assay (Fig 5). Interestingly, there were no significant differences in titers produced by the two viruses in any of the organs selected for study at any time point tested. This finding raises the possibility that in the context of the reassortant viruses, σ3-Y354H enhances lethality via a different mechanism than the kinetic replication advantage observed following intramuscular inoculation (Fig 2). To test the stability of the σ3-Y354H mutation during *in vivo* replication, we isolated viral clones from cardiac tissue of mice infected with either T1L/T3Dμ1σ3 or T1L/T3Dμ1σ3Y354H (three mice per strain). We sequenced the S4 gene segments of five viral clones from each mouse infected with T1L/T3Dμ1σ3 and 10 clones per mouse from those infected with T1L/T3Dμ1σ3Y354H. No reversions or second-site mutations were observed for either reassortant strain.

### The Y354H mutation in σ3 exacerbates reovirus-inducted myocarditis

**Fig 6:**
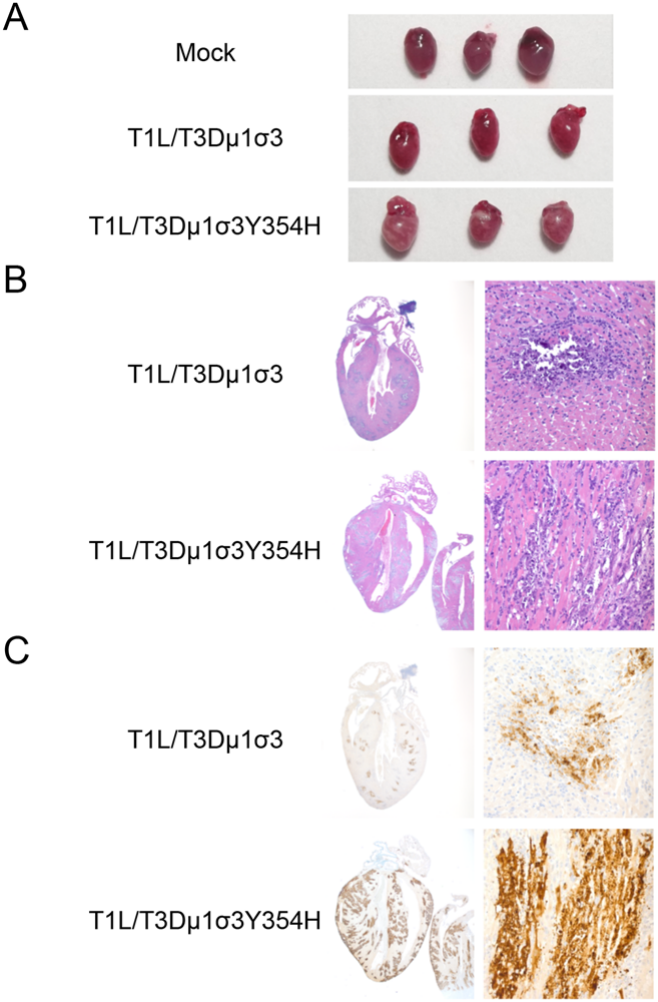
Reovirus strain T1L/T3Dμ1σ3Y354H causes pronounced myocarditis. Newborn C57BL/6J mice were inoculated perorally with 10^3^ PFU of either T1L/T3Dμ1σ3 or T1L/T3Dμ1σ3Y354H. On day 8 post-inoculation, mice were euthanized, and hearts were excised and photographed (A). Cardiac tissue was fixed in formalin, embedded in paraffin, sectioned, and stained with H&E (B) or reovirus-specific polyclonal antiserum (C). Images are shown at 20X (left) and 400X (right) magnification.

Type 1 reovirus strains induce myocarditis in newborn mice following peroral inoculation [70–72]. To investigate whether enhanced lethality in mice inoculated with T1L/T3Dμ1σ3Y354H was due to reovirus-induced myocarditis, we inoculated newborn mice with 10^3^ PFU of either reassortant strain T1L/T3Dμ1σ3 or T1L/T3Dμ1σ3Y354H and excised hearts of infected mice 8 days post-inoculation. The dose used for these experiments was decreased from 10^4^ to 10^3^ PFU to ensure that no mice succumbed to lethal infection before the day 8 time point. Hearts of mice inoculated with T1L/T3Dμ1σ3Y354H displayed markedly greater gross pathology than hearts from T1L/T3Dμ1σ3-infected animals (Fig 6A). Histological examination revealed multifocal areas of myocardial injury throughout both atria and ventricles and the interventricular septum (Figs 6B and S2). These areas consisted of myocyte necrosis with persistence of macrophages but no neutrophils. Microcalcification also was observed in most of these areas, as was the presence of apoptotic nuclei. Foci of histological injury were more widespread in cardiac tissue from mice infected with T1L/T3Dμ1σ3Y354H than from those infected with the wild-type reassortant. Despite equivalent viral titers, hearts of mice infected with T1L/T3Dμ1σ3Y354H displayed greater reovirus antigen distribution than those from T1L/T3Dμ1σ3-infected mice (Figs 6C and S2, S3). This finding is consistent with previous reports demonstrating that myocarditic and non-myocarditic reovirus strains replicate to comparable titers [73] and highlights the possibility that myocytes undergo necrosis more rapidly in animals infected with the mutant virus and thus do not allow completion of the viral replication cycle. Therefore, introduction of σ3-Y354H into the genetic background of T1L/T3Dμ1σ3 results in exaggerated myocarditis, which is likely responsible for the differences in survival following peroral challenge with the reassortant viruses.

### T1L/T3Dμ1σ3Y354H induces enhanced inflammatory cytokine expression in cardiac tissue

**Fig 7:**
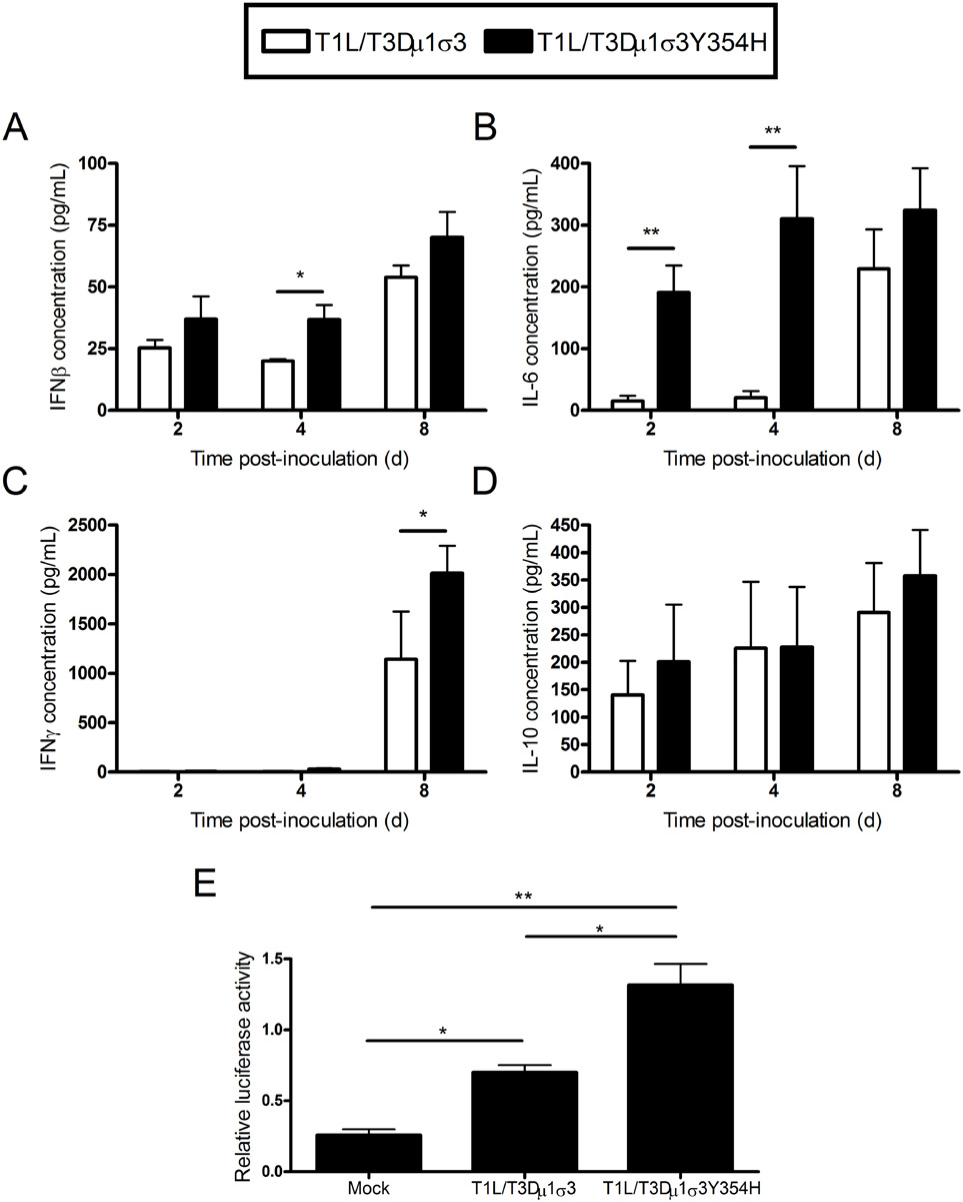
Elevated cytokine levels following infection of mice with T1L/T3Dμ1σ3 and T1L/T3Dμ1σ3Y354H. (A-D) Newborn C57BL/6J mice were inoculated perorally with 10^3^ PFU of either T1L/T3Dμ1σ3 or T1L/T3Dμ1σ3Y354H. At days 2, 4, and 8 post-inoculation, mice were euthanized, and hearts were excised, frozen at -80°C, thawed, and homogenized in PBS. Levels of IFNβ (A), IFNγ (B), IL-6 (C), and IL-10 (D) in heart homogenates were quantified by ELISA. Results are expressed as mean cytokine levels for 4–7 animals per time point. (E) Human embryonic kidney cells (293-T) were cotransfected with a plasmid encoding *Renilla* luciferase as a transfection control and either a PGL firefly luciferase reporter plasmid under the control of the IFN-sensitive reporter element (ISRE) or a PGL-basic vector as a general transcription control. Cells were incubated for 24 h and inoculated with the viruses shown at an MOI of 100 PFU/cell. Luciferase activity was quantified 24 h post-inoculation. Values for cells expressing the ISRE reporter were normalized to the corresponding values for the PGL-basic control vector as a transcription control. Data represent an experiment conducted twice in triplicate. Error bars indicate standard errors of the mean. *, *P* < 0.05, **, *P* < 0.01 as determined by Student’s *t* test.

Reovirus-mediated myocarditis is modulated by innate immune responses [70–72], suggesting that the severe cardiac damage induced by T1L/T3Dμ1σ3Y354H might be attributable to altered innate immune responses in cardiac tissue. To test this hypothesis, we quantified cytokine expression in cardiac tissue homogenates following infection of mice with either T1L/T3Dμ1σ3 or T1L/T3Dμ1σ3Y354H. Mice were inoculated perorally with either of the two reassortant strains, hearts were excised at days 2, 4, and 8 post-inoculation, and concentrations of interferon-β (IFNβ), IFNγ, IL-6, and IL-10 in cardiac tissue homogenates were determined by ELISA. Hearts of mice inoculated with T1L/T3Dμ1σ3Y354H displayed higher levels of IFNβ and IL-6 at early time-points as well as increased IFNγ at day 8 in comparison to hearts from animals inoculated with the wild-type reassortant (Fig 7A-C). No differences in IL-10 induction were observed (Fig 7D). Therefore, the enhanced tissue damage induced by T1L/T3Dμ1σ3Y354H correlates with an exaggerated cardiac innate response. Concordantly, T1L/T3Dμ1σ3Y354H induced greater type 1 IFN pathway stimulation in cultured cells as assessed using an ISRE luciferase reporter plasmid compared with that induced by wild-type T1L/T3Dμ1σ3 (Fig 7E). Taken together, these data suggest that capsid instability enhances cytokine signaling, perhaps because of more rapid stimulation of innate immune signal-transduction pathways within cells, even when matched for cumulative viral load across the tissue.

To determine whether the relationship between capsid stability and inflammatory cytokine production in the heart is specific to the genetic background of the infecting viral strain, mice were inoculated intramuscularly with either T3D or T3D-σ3Y354H, hearts were excised at days 2, 4, and 8 post-inoculation, and concentrations of interferon-β (IFNβ), IL-6, IFNγ, and IL-10 in cardiac tissue homogenates were determined by ELISA. Levels of IFNβ or IL-6 in infected cardiac homogenates were modest and did not differ between the two strains (S4A-S4B Fig). Hearts of mice inoculated with T3D-σ3Y354H displayed slightly higher levels of IFNγ and significantly higher levels of IL-10 at day 8 in comparison to hearts from animals inoculated with wild-type T3D (S4C-S4D Fig).

We wondered whether the effect of capsid stability on virulence was dependent on inoculation route. As T3D does not survive the intestinal environment due to cleavage of its σ1 attachment molecule [66,67], we inoculated newborn mice intramuscularly with 10^4^ PFU of either T1L/T3Dμ1σ3 or T1L/T3Dμ1σ3Y354H. We found no significant difference in mortality between wild-type and mutant viruses (S5 Fig), and titers of T1L/T3Dμ1σ3 exceeded those of T1L/T3Dμ1σ3Y354H at several sites of secondary replication (S6 Fig). However, in comparison to T1L/T3Dμ1σ3, T1L/T3Dμ1σ3Y354H was associated with increased levels of IL-6 in heart homogenates (S7 Fig), although the observed differences were not statistically significant. These findings suggest that linkage between capsid stability and viral virulence is influenced by inoculation route, yet a capsid-destabilizing mutation is associated with increased cytokine levels in organs targeted by reovirus independent of the method of inoculation.

### The Y354H mutation in σ3 increases the frequency of host-to-host transmission and severity of disease in uninfected littermates

**Fig 8:**
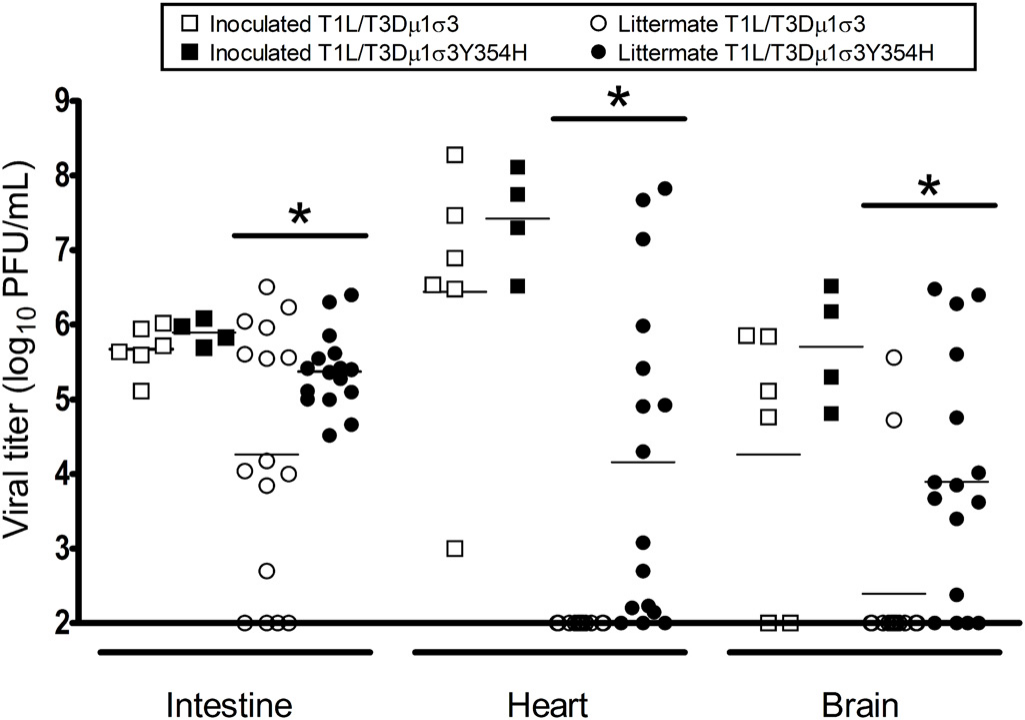
The σ3-Y354H mutation is associated with higher viral loads after transmission of reovirus between littermates. Two newborn C57BL/6J mice from a litter of eight animals were inoculated perorally with 10^4^ PFU of either T1L/T3Dμ1σ3 (black) or T1L/T3Dμ1σ3Y354H (white). The inoculated mice (squares) were placed with their uninoculated littermates (circles) and housed together. Eight days later, inoculated mice and uninoculated littermates were euthanized, intestine, heart, and brain were excised, and viral titers were determined by plaque assay. Results are expressed as viral titers for each animal assayed. *, *P* < 0.05 as determined by Mann-Whitney test in comparison to T1L/T3Dμ1σ3.

Given the diminished capsid stability imposed by σ3-Y354H [60], we hypothesized that σ3-Y354H-containing viruses might spread less efficiently between hosts due to diminished viability or decreased persistence on fomite surfaces. To test this hypothesis, we divided newborn mice into litters of eight animals each, inoculated two animals from each litter with 10^4^ PFU of either T1L/T3Dμ1σ3 or T1L/T3Dμ1σ3Y354H, and replaced the infected pups with their uninfected littermates. Eight days post-inoculation, both inoculated and uninoculated littermates were euthanized, intestine, heart, and brain were excised, and viral titers in those organs were determined by plaque assay. Consistent with our previous findings, viral titers in animals inoculated with T1L/T3Dμ1σ3 and T1L/T3Dμ1σ3Y354H were comparable, albeit slightly higher in mutant-infected mice. However, titers in the intestine, heart, and brain of naïve littermates housed with animals inoculated with T1L/T3Dμ1σ3Y354H were significantly higher than those in littermates of animals inoculated with T1L/T3Dμ1σ3 (Fig 8). This finding suggests that σ3-Y354H is associated with increased littermate transmission and increased replication in newly infected pups.

To determine whether the observed differences in transmission were attributable to differences in viral shedding, we quantified the titer of infectious virus in fecal matter of infected animals. Mice were inoculated perorally with 10^4^ of either T1L/T3Dμ1σ3 or T1L/T3Dμ1σ3Y354H. Stool pellets were recovered from infected mice on days 2, 4, and 8 post-inoculation, suspended in PBS, and processed for viral titer determination by plaque assay. No significant differences were observed in the fecal titers of the reassortant viruses, indicating that animals infected with T1L/T3Dμ1σ3Y354H do not shed higher titers of virus than those infected with the wild-type reassortant (S8 Fig). The higher titers observed for T1L/T3Dμ1σ3Y354H in uninoculated littermates are thus less likely to be the result of exposure to a higher initial dose shed from inoculated animals but rather due to enhanced replication within recipient animals.

## Discussion

Achieving the optimal balance between capsid stability and instability is a challenge faced by all viruses, yet the influence of capsid stability on disease pathogenesis is not well understood. Previous studies suggest that capsid stability contributes to but is not the sole determinant of poliovirus virulence in mice [74]. In this study, we used reovirus to investigate the effect of capsid instability on viral disease. To productively infect target cells, reovirus must undergo stepwise disassembly mediated by host proteases [49,75,76]. The initial step in this uncoating process is the proteolytic cleavage of outer-capsid protein σ3. A single mutation in the σ3 C-terminus, Y354H, increases the rate of σ3 proteolysis and confers viral resistance to inhibitors of acid-dependent proteases such as E64 and ammonium chloride [77]. The σ3-Y354H phenotype is not protease-specific; rather, σ3-Y354H induces a structural alteration that accelerates attack by a variety of proteases [77]. Viruses with the σ3-Y354H mutation lose titer more rapidly when exposed to elevated temperature than do those with native σ3, indicating that σ3-Y354H reduces biophysical capsid stability [60] (S1 Fig). Additionally, σ3-Y354H is largely absent from primary reovirus isolates, supporting the hypothesis that σ3-Y354H imposes some type of fitness cost [61]. We employed the capsid-destabilizing effect of the σ3-Y354H polymorphism to determine the role of capsid stability in reovirus-mediated disease.

Contrary to our initial hypothesis, we found that T3D-σ3Y354H displayed significantly enhanced virulence in newborn mice compared with wild-type T3D. Strain T3D is neurotropic, and mice infected with either T3D or T3D-σ3Y354H developed neurological findings, including paralysis and seizures. We found that T3D-σ3Y354H displayed significantly enhanced virulence in newborn mice compared with T3D. In addition, T3D-σ3Y354H replicated to higher titers at days 2 and 4 post-inoculation in the hindlimb muscle as well as at several sites of secondary replication, including the liver and heart. As infection progressed, the two viruses reached equivalent peak titers in all tissues tested. This plateau effect likely reflects the limit of viral replication supported by a given organ and has been observed in other studies of reovirus pathogenesis [28,32,78–80]. Thus, for strain T3D, the σ3-Y354H mutation confers a replication advantage early in infection, allowing more rapid dissemination and seeding of secondary sites of replication. The cumulative burden of viral replication over time, particularly in the central nervous system, likely accounts for the enhanced lethality of T3D-σ3Y354H.

The natural route of reovirus transmission is thought to be fecal-oral. However, T3D is not infectious when delivered perorally because the T3D σ1 attachment protein is hypersensitive to proteolysis by digestive enzymes [66,67]. Therefore, we engineered reassortant reovirus strains containing the T3D μ1 and σ3 proteins, both with and without the σ3-Y354H mutation, in an otherwise T1L genetic background, which infects efficiently following peroral inoculation. In newborn mice, the σ3-Y354H-containing virus also was substantially more virulent in comparison to the virus expressing wild-type σ3, strengthening the conclusion that σ3 is a reovirus virulence determinant. The Y354H mutation was stable *in vivo*, suggesting that differences in virulence are not attributable to reversion of the mutation. We note that both reassortant strains are virulent in comparison to other non-neurotropic reovirus strains. Although the LD_50_ values of the two viruses were not formally determined, we observed approximately 50 percent mortality in animals inoculated perorally with 10^4^ PFU of the wild-type reassortant and approximately 90 percent mortality in those inoculated perorally with the reassortant containing σ3-Y354H. Therefore, the combination of a T3D outer capsid with a T1L core results in particularly virulent strains of reovirus, an effect exacerbated by σ3-Y354H.

We find it noteworthy that viral loads in organs of mice infected with either T1L/T3Dμ1σ3 or T1L/T3Dμ1σ3Y354H were essentially equivalent, even at very early time points post-inoculation. The reassortant viruses encode a T1L σ1 attachment protein and, thus, are not neurotropic [29,62]. However, there was overt cardiac tissue injury and dystrophic myocardial changes in mice inoculated with either reassortant strain, although the effect was much more substantial in mice infected with the σ3-Y354H reassortant.

The precise mechanism by which σ3-Y354H enhances cardiac injury is unclear. Despite increased gross pathology, myocyte necrosis, and viral antigen staining, viral titers were comparable between the wild-type and σ3-Y354H-containing reassortants. This finding is consistent with previous evidence that reoviruses inducing dramatic cardiac damage can produce titers in cardiac tissue similar to those produced by reoviruses inducing almost undetectable damage [73]. The presence of increased viral antigen in the absence of increased viral titer suggests that the immunohistochemistry may detect increased viral protein production in apoptotic or necrotic cells in the σ3-Y354H-infected hearts that will not produce viable progeny virus. However, while no significant differences in viral titer were observed, viral loads in σ3-Y354H-infected hearts trended higher than those in wild-type-infected hearts, suggesting that replication of σ3-Y354H-containing virus might be more efficient in cardiac tissue.

We hypothesized that σ3-Y354H might alter the host response to reovirus infection in the T1L genetic background, accounting for the enhanced tissue damage. Induction of IFN is protective against reovirus-induced cardiac injury [81]. Interestingly, we observed increased induction of proinflammatory cytokine IL-6 at early time points in the hearts of mice infected with T1L/T3Dμ1σ3Y354H in comparison to the wild-type reassortant. Additionally, σ3-Y354H-containing virus induced increased levels of both IFNβ and IFNγ in the hearts of infected animals at both early and late time points. Furthermore, an ISRE luciferase reporter assay indicates that σ3-Y354H increases type 1 IFN-mediated signaling *in vitro*, suggesting that a property linked to σ3 during viral entry is responsible for enhanced cytokine production. The increased induction of proinflammatory cytokines may reflect faster replication kinetics that overwhelm the innate cardiac protective response. Such cytokines may directly mediate injury to the heart muscle or potentiate downstream effects leading to cardiac myocyte damage. Our results indicate that the rate of viral particle disassembly modulates the myocarditic capacity of reovirus *in vivo*.

The relationship between capsid stability and virulence appears to be somewhat influenced by viral strain, mechanism of dissemination, and route of inoculation; yet, in all tested scenarios, the σ3-Y354H mutation was associated with higher levels of cytokine production in the heart. Serotype 3 reovirus, including strain T3D, spreads via neural routes [30,31]. Following intramuscular inoculation, T3Dσ3-Y354H displayed enhanced spread to sites of secondary replication. Strains containing a serotype 1 σ1, such as the reassortant strains T1L/T3Dμ1σ3 and T1L/T3Dμ1σ3Y354H spread hematogenously [27,28]. Following peroral inoculation, the reassortant containing σ3-Y354H displayed enhanced inflammatory cytokine production, myocarditis, and lethality. Thus, in mice inoculated intramuscularly or perorally with reovirus strains that spread via neural or hematogenous routes, respectively, capsid stability is a virulence determinant.

In contrast to the differences in virulence displayed by T1L/T3Dμ1σ3 and T1L/T3Dμ1σ3Y354H following peroral inoculation, these strains were comparably virulent following intramuscular inoculation. Additionally, T1L/T3Dμ1σ3 produced higher titers at sites of secondary replication subsequent to intramuscular inoculation than did T1L/T3Dμ1σ3Y354H. Nonetheless, despite producing lower titers in the heart, T1L/T3Dμ1σ3Y354H infection was associated with increased cytokine production. Collectively, these findings suggest that the effect of σ3-Y354H in the genetic background of T1L is most manifest following peroral inoculation, perhaps as a consequence of the proteolytic milieu in the intestine. Accelerated kinetics of virion-to-ISVP conversion afforded by the capsid-destabilizing Y354H mutation in σ3 may allow more efficient establishment of infection in the intestine by the mutant strain for dissemination to target tissues.

Since σ3-Y354H imposes no obvious fitness penalty *in vivo*, we considered the possibility that the mutation impairs host-to-host transmission. Spread of reovirus to naïve hosts by the fecal-oral route requires some degree of environmental persistence, and σ3-Y354H diminishes the thermostability of the viral particle [60] (S1 Fig). Thus, it seemed plausible that destabilizing mutations in the reovirus outer capsid reduce the duration of persistence of infectious virus particles, limiting their spread. To determine whether the σ3-Y354H mutation impairs host-to-host transmission, we compared viral titers in the intestines, hearts, and brains of naïve littermates housed for eight days with mice inoculated with either of the reassortant strains. Surprisingly, σ3-Y354H enhanced host-to-host spread in this model, as uninoculated littermates of animals infected with the σ3-Y354H reassortant had significantly higher viral loads in all organs tested in comparison to littermates of mice infected with the wild-type reassortant. Viral titers in stool did not differ significantly between the two reassortants (S8 Fig), indicating that σ3-Y354H does not facilitate increased viral shedding from the intestine of inoculated animals and, furthermore, that uninoculated littermates were exposed to comparable doses of shed virus. These findings support the hypothesis that σ3-Y354H in the T1L genetic background enhances the specific infectivity of reovirus particles in the intestine.

Given the effects of σ3-Y354H on reovirus replication and transmission, it remains unclear why the mutation is absent from circulating reovirus strains except in the presence of compensatory second-site changes [60]. It is possible that our littermate-transmission assay may not accurately reflect natural reovirus spread, and a more strenuous test of reovirus transmission might identify fitness deficits imposed by diminished capsid stability. For example, as reovirus is a nonenveloped virus and fairly stable in the environment, it is possible that capsid stability alters fomite transmission. Another possibility is that reovirus has evolved to be a mild, often asymptomatic pathogen, and the induction of more severe disease may be maladaptive in some way. Severely ill hosts may shed progeny virus for shorter intervals, or exaggerated immune responses might limit viral replication. We find it remarkable that while nearly all adults are seropositive for reovirus [82], severe disease is rarely reported. It is difficult to test these hypotheses without greater understanding of the natural ecology of reovirus infection and transmission.

In this study, we found that reducing the stability of the reovirus capsid enhances reovirus-mediated disease, an effect that is penetrant in different strains of reovirus and via different routes of inoculation. The introduction of the σ3-Y354H polymorphism results in reovirus strains with increased pathogenicity, enhanced replication *in vivo*, and increased capacity for host-to-host spread. This work identifies reovirus outer-capsid protein σ3 as a new determinant of virulence and more broadly suggests that capsid stability influences the pathogenesis of nonenveloped viruses.

## Materials and Methods

### Cells and viruses

Spinner-adapted murine L929 cells were grown in either suspension or monolayer cultures in Joklik’s modified Eagle’s minimal essential medium (SMEM; Lonza, Walkersville, MD) supplemented to contain 5% fetal bovine serum (Invitrogen; Carlsbad, CA), 2 mM L-glutamine (Invitrogen), 100 U of penicillin per mL, 100 U of streptomycin per ml (Invitrogen), and 0.25 μg of amphotericin per ml (Sigma-Aldrich; St. Louis, MO). BHK-T7 cells were grown in Dulbecco’s modified Eagle’s minimal essential medium (Invitrogen) supplemented to contain 5% fetal bovine serum, 2 mM L-glutamine, 2% MEM amino acid solution (Invitrogen), and 1 mg geneticin per ml (Invitrogen). Human 293T cells were maintained in Dulbecco’s modified Eagle’s minimal essential medium supplemented to contain 10% fetal bovine serum, 2 mM L-glutamine, 100 U of penicillin per mL, 100 U of streptomycin per mL, and 0.25 μg of amphotericin per ml.

Recombinant strain (rs) T3D is a stock generated by plasmid-based reverse genetics from cloned T3D cDNAs [83]. The engineered reovirus mutant T3D-σ3Y354H and the reassortant viruses T1L/T3Dμ1σ3 and T1L/T3Dμ1σ3Y354H were generated as described [59].

### Infection of mice

C57BL/6J mice were obtained from Jackson Laboratory. Two-to-three-day-old mice were inoculated intramuscularly or perorally with purified reovirus diluted in PBS. Intramuscular inoculations (10 μl) were delivered into the left hindlimb (hamstring muscle) using a Hamilton syringe and 30-gauge needle. Peroral inoculations (50 μl) were administered using a tuberculin slip tip syringe, 30-gauge needle, and Intramedic PE-10 polyethylene tubing (BD Biosciences) [27,84]. For analysis of viral virulence, mice were monitored for signs of disease for 21 days post-inoculation. Mice were euthanized when found to be moribund (defined by rapid or shallow breathing, lethargy, or paralysis). Data from these experiments are reported as “percent survival,” although death was not used as an endpoint. For analysis of virus replication, mice were euthanized at defined intervals post-inoculation, and organs were excised into 1 ml of PBS and homogenized by freezing, thawing, and sonication. Intestines were transected proximally at the gastroduodenal junction and distally at the rectum before homogenization in 1 mL of PBS. Viral titers in organ homogenates were determined by plaque assay using L929 cells [85]. For quantification of fecal viral titers, stool pellets were collected at defined intervals post-inoculation, suspended in 1 mL of PBS, and homogenized by freezing, thawing, and sonication. Viral titers were determined by plaque assay. For immunohistochemical analysis, mice were euthanized at defined intervals post-inoculation, and organs were excised and fixed overnight in 10% formalin. Fixed organs were embedded in paraffin, and 6-μm histological sections were prepared. Sections were processed for hematoxylin and eosin staining and detection of reovirus protein using polyclonal reovirus-specific antiserum as described [27].

For littermate transmission studies, newborn mice were divided into litters of eight animals. Two animals per litter were inoculated perorally and replaced into original cages with dams and uninoculated littermates. Both inoculated and uninoculated animals were euthanized 8 days post-inoculation, and viral titers in various organs were determined by plaque assay.

### Ammonium chloride sensitivity assay

Confluent monolayers of L929 cells (approximately 2 × 10^5^ cells/well) in 24-well plates (Costar) were incubated at 37°C for 1 h with SMEM supplemented to contain 0 to 10 mM ammonium chloride (Sigma). The medium was removed, and cells were incubated with second- or third-passage virus stocks at a multiplicity of infection (MOI) of 25 plaque-forming units (PFU) per cell at 4°C for 1 h. The inoculum was removed, cells were washed with PBS, and 1 ml of fresh SMEM supplemented to contain 0 to 10 mM ammonium chloride was added. Cells were incubated at 37°C for 18 h, fixed with methanol at -20°C for 30 min, and stained with rabbit reovirus-specific antiserum [86], followed by Alexa 488-conjugated goat anti-rabbit secondary antibody (Invitrogen) and 4′,6-diamidino-2-phenylindole (DAPI, Invitrogen). Infected cells were visualized using fluorescence microscopy. Total cell number was determined by DAPI staining and quantified using ImageJ software (Rasband, WS; NIH, Bethesda, Maryland).

### Heat resistance of reovirus virions

Purified reovirus virions (2 × 10^8^ particles/mL) were incubated at 55°C for 60 min. Aliquots were removed at 15-min intervals and placed on ice (58). Viral titers were quantified by plaque assay.

### Cytokine expression assay

Two-to-three-day-old mice were inoculated perorally with purified reovirus diluted in PBS. At 2, 4, and 8 days post-inoculation, mice were euthanized and hearts were excised, frozen at -80°C, thawed, and homogenized by sonication in 1 mL of PBS. Concentrations of IFNβ (BioLegend), IFNγ, IL-6, and IL-10 (Ready-Set-Go!, eBioscience, San Diego, California) protein were determined in heart homogenates by ELISA according to manufacturers’ instructions.

### Luciferase reporter assay

Dual-luciferase reporter assays were performed as described [87]. 293T cells cultivated in 24-well plates were transfected with 0.2 μg of a reporter plasmid (Stratagene) that expresses firefly luciferase under the control of an IFN-sensitive promoter (ISRE) or a PGL-basic plasmid as a control (Promega). Each well was transfected with 0.2 μg of pRenilla-luc, which constitutively expresses *Renilla* luciferase, as a loading control. Transfections were carried out using Fugene (Roche). Following 24 h incubation, cells were adsorbed with reovirus in serum-free medium at room temperature for 1 h and incubated in complete medium for 24 h. Luciferase reporter activity was quantified using the dual luciferase assay kit (Promega) according to the manufacturer’s instructions.

### Statistical analysis

A log-rank test was used to compare survival frequency of mice inoculated with different reovirus strains. For experiments in which viral titers were determined in an organ or percent of infected cells was determined by indirect immunofluorescence, a Mann-Whitney test was used to calculate two-tailed *P* values [88]. For experiments involving cytokine induction *P* values were calculated using Student’s *t* test. *P* values of < 0.05 were considered to be statistically significant. Statistical analyses were performed using Prism software (GraphPad Software, San Diego, California).

### Ethics statement

Animal husbandry and experimental procedures were performed in accordance with Public Health Service policy and approved by the Vanderbilt University School of Medicine Institutional Animal Care and Use Committee. The experiments described herein were performed under institutional protocol M/07/159 in accordance with the Guide for the Care and Use of Laboratory Animals, Eighth Edition (National Academies Press) and the American Veterinary Medical Association Guidelines for the Euthanasia of Animals: 2013 Edition (AVMA).

In the course of conducting this study, we found that a reovirus reassortant strain with a capsid-destabilizing mutation unexpectedly displayed enhanced lethality and transmissibility in mice. As the data were being collected, we consulted with representatives of the Vanderbilt University School of Medicine Division of Animal Care and Institutional Biosafety Committee for guidance about the conduct of the experiments. We also discussed this work with program staff at the National Institute of Allergy and Infectious Diseases. It was concluded that the ABSL-2 biosafety protocols employed in this research were appropriate.

## Supporting Information

S1 Fig Resistance of reovirus strains to heat inactivation. Purified virions were diluted in virion storage buffer to a concentration of 2 × 10^8^ particles/mL and incubated at 55°C for 60 min. At 15-min intervals, samples were removed and placed on ice. Titers were determined by plaque assay. Results are presented as the percentage of mean viral titer of untreated samples per interval of incubation for triplicate experiments. Error bars represent standard deviations. *, *P* < 0.05 as determined by two-way ANOVA.
(TIF)

S2 Fig Reovirus-induced cardiac injury low-magnification. Newborn C57BL/6J mice were inoculated perorally with 10^3^ PFU of either T1L/T3Dμ1σ3 (A, B, E, F) or T1L/T3Dμ1σ3Y354H (C, D, G, H). On day 8, mice were euthanized, and hearts were excised. Cardiac tissue was fixed in formalin, embedded in paraffin, sectioned, and stained with H&E (A-D) or reovirus-specific polyclonal antiserum (E-H). Images are shown at 20X magnification.
(TIF)

S3 Fig Reovirus-induced cardiac injury high-magnification. Newborn C57BL/6J mice were inoculated perorally with 10^3^ PFU of either T1L/T3Dμ1σ3 (A, B, E, F) or T1L/T3Dμ1σ3Y354H (C, D, G, H). On day 8, mice were euthanized, and hearts were excised. Cardiac tissue was fixed in formalin, embedded in paraffin, sectioned, and stained with H&E (A-D) or reovirus-specific polyclonal antiserum (E-H). Images are shown at 400X magnification.
(TIF)

S4 Fig Cytokine levels in cardiac tissue of mice inoculated intramuscularly with T3D and T3Dσ3Y354H. Newborn C57BL/6J mice were inoculated intramuscularly with 10^5^ PFU of either T3D or T3D-Y354H. At days 2, 4, and 8 post-inoculation, mice were euthanized, and hearts were excised, frozen at -80°C, thawed, and homogenized in PBS. Levels of IFNβ (A), IFNγ (B), IL-6 (C), and IL-10 (D) in heart homogenates were quantified by ELISA. Results are expressed as mean cytokine levels for 6–9 animals per time point. Error bars indicate standard errors of the mean. ***, *P* < 0.001 as determined by Student’s *t* test.
(TIF)

S5 Fig T1L/T3Dμ1σ3 and T1L/T3Dμ1σ3Y354H are virulent following intramuscular inoculation. Newborn C57BL/6J mice were inoculated intramuscularly with 10^4^ PFU of either T1L/T3Dμ1σ3 or T1L/T3Dμ1σ3Y354H. Mice (n = 14 and 12 for T1L/T3Dμ1σ3 and T1L/T3Dμ1σ3Y354H, respectively) were monitored for survival for 21 days. Differences between T1L/T3Dμ1σ3 and T1L/T3Dμ1σ3Y354H are not significant as determined by log-rank test.
(TIF)

S6 Fig Viral loads in mice infected intramuscularly with T1L/T3Dμ1σ3 and T1L/T3Dμ1σ3Y354H. Newborn C57BL/6J mice were inoculated in the left hindlimb with 10^4^ PFU of either T1L/T3Dμ1σ3 or T1L/T3Dμ1σ3Y354H. At days 2 (A), 4 (B), and 8 (C) post-inoculation, animals were euthanized, left hindlimb muscle, heart, and brain were excised, and viral titers in organ homogenates were determined by plaque assay. Results are expressed as mean viral titers for 4 to 5 animals for each time point. Error bars indicate standard errors of the means. *, *P* < 0.05 as determined by Mann-Whitney test in comparison to T1L/T3Dμ1σ3.
(TIF)

S7 Fig Cytokine levels in cardiac tissue of mice infected intramuscularly with T1L/T3Dμ1σ3 and T1L/T3Dμ1σ3Y354H. Newborn C57BL/6J mice were inoculated in the left hindlimb with 10^4^ PFU of either T1L/T3Dμ1σ3 or T1L/T3Dμ1σ3Y354H. At days 2, 4, and 8 post-inoculation, mice were euthanized, and hearts were excised, frozen at -80°C, thawed, and homogenized in PBS. Levels of IFNβ (A), IL-6 (B), and IFNγ (C) in heart homogenates were quantified by ELISA. Results are expressed as mean cytokine levels for 4–5 animals per time point. Error bars indicate standard errors of the mean. Differences between T1L/T3Dμ1σ3 and T1L/T3Dμ1σ3Y354H are not significant as determined by Student’s *t* test.
(TIF)

S8 Fig Fecal titers are equivalent following inoculation with wild-type and σ3-Y354H- containing reassortants. Newborn C57BL/6J mice inoculated perorally with 10^4^ PFU of either T1L/T3Dμ1σ3 or T1L/T3Dμ1σ3Y354H. On days 2, 4, and 8 post-inoculation, viral titers in stool samples were quantified by plaque assay. Results are expressed as viral titers for 3–4 mice per time point per virus. Differences in viral titer are not statistically significant by Student’s *t* test.
(TIF)
