# Supplementary figures and images for: Diminished Reovirus Capsid Stability Alters Disease Pathogenesis and Littermate Transmission
Source: PLoS Pathog. 2015 Mar 4;11(3):e1004693. doi: 10.1371/journal.ppat.1004693 (PMC4349883; doi:10.1371/journal.ppat.1004693)

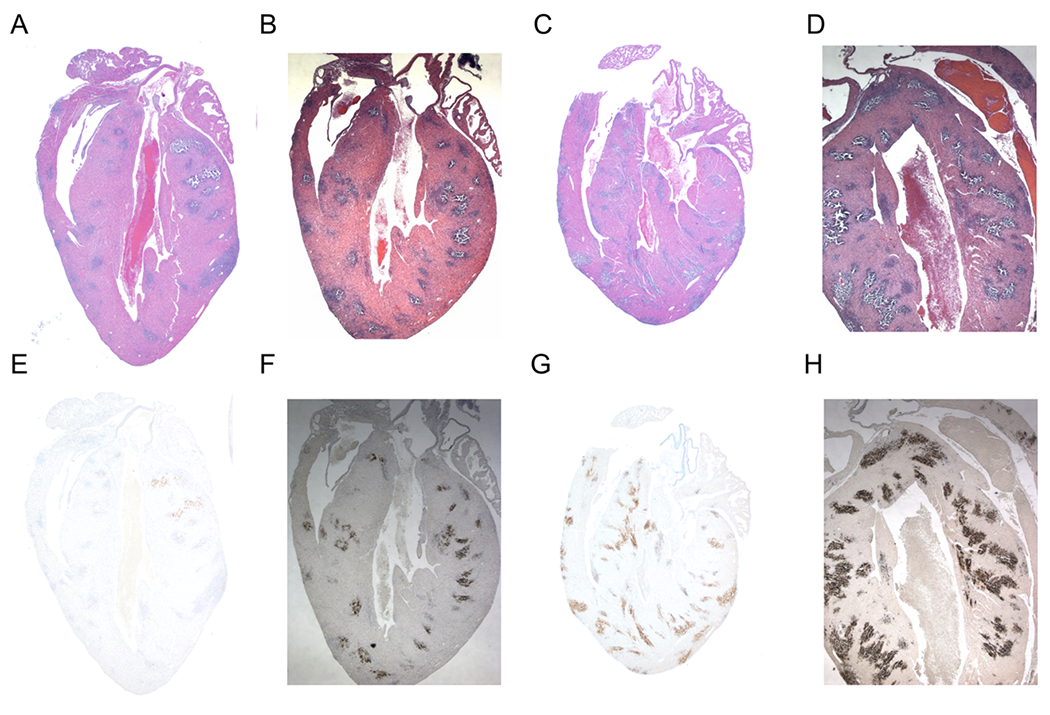

Supplement: S2 Fig — Newborn C57BL/6J mice were inoculated perorally with 103 PFU of either T1L/T3Dμ1σ3 (A, B, E, F) or T1L/T3Dμ1σ3Y354H (C, D, G, H). On day 8, mice were euthanized, and hearts were excised. Cardiac tissue was fixed in formalin, embedded in paraffin, sectioned, and stained with H&E (A-D) or reovirus-specific polyclonal antiserum (E-H). Images are shown at 20X magnification. (TIF) [file ppat.1004693.s002.tif]

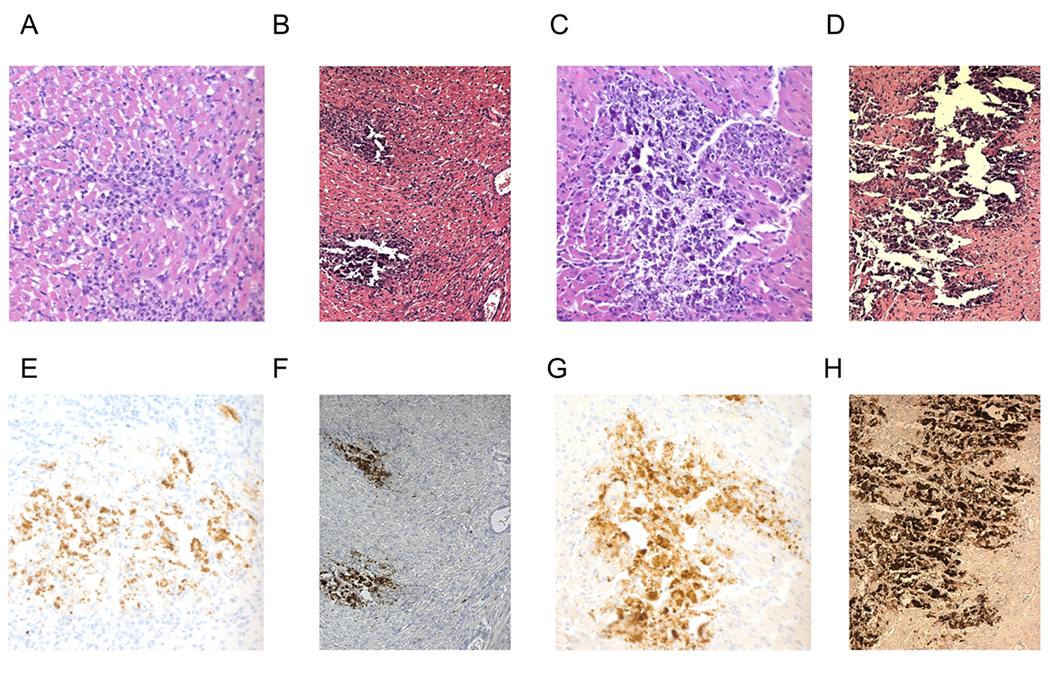

Supplement: S3 Fig — Newborn C57BL/6J mice were inoculated perorally with 103 PFU of either T1L/T3Dμ1σ3 (A, B, E, F) or T1L/T3Dμ1σ3Y354H (C, D, G, H). On day 8, mice were euthanized, and hearts were excised. Cardiac tissue was fixed in formalin, embedded in paraffin, sectioned, and stained with H&E (A-D) or reovirus-specific polyclonal antiserum (E-H). Images are shown at 400X magnification. (TIF) [file ppat.1004693.s003.tif]
